# Supplementary material for: Recommendations for 24-Hour Movement Behaviours in Adults with Asthma: A Review of Current Guidelines
Source: Int J Environ Res Public Health. 2020 Mar 10;17(5):1789. doi: 10.3390/ijerph17051789 (PMC7084595; doi:10.3390/ijerph17051789)
Supplement: Supplementary file 1 [file ijerph-17-01789-s001.pdf]

SUPPLEMENTARY MATERIALS

Table S1. Recommendations and Strategies (verbatim) pertaining to Physical Activity from Clinical Practice Guidelines for Asthma.

CPG		Target	Recommendations around frequency, intensity, time or type				Verbatim text	SB
			F	I	Time	Type		
India	2005	A + P	-	-	-	Yogic/ relaxation exercises	*Recommendation*<br>"Many of these remedies especially the use of foods such as the fresh green vegetables and fruits, yogic and relaxation exercises improve the quality of life and can be incorporated as healthy lifestyle measures." p 333<br><br>*Strategy*<br>"...the goals as defined by the Global Initiative for Asthma (GINA) would include: No limitation of physical activity, minimal (ideally none) symptoms during exercise." p 318<br><br>"The restriction of physical exercise is not advisable. Rather asthmatics should be encouraged to participate in exercises. Short acting beta-2 agonists should be used prior to anticipated exercise in a patient with exercise-induced asthma to alleviate symptoms." p 332	*No*
Japan	2014	A	-	-	-	-	*Strategy*<br>"LTRAs are useful for long-term management of patients with asthma complicated by allergic rhinitis, exercise-induced asthma (EIA), and AIA. Generally, LTRAs alone are less effective compared with low doses of ICSs, whereas the effects of LTRAs plus ICSs are reported to be the same as those of LABAs plus ICSs in steroid-naïve asthmatic patients." p 305<br><br>"Prevention of exercise-induced asthma: Inhaled β2-stimulants are superior to other asthma drugs owing to their bronchodilatory action; they prevent bronchoconstriction induced by EIA. LTRAs are also useful and comparable or superior to LABAs for the prevention of EIA. EIA should be prevented by long-term management with controller drugs and inhalation of β2-stimulants before exercise." p 319	*No*

							"Prevention of asthma in athletes: To prevent exercise-induced attack of asthma, athletes need to be treated with SABAs 10 to 15 min prior to exercise. If asthma symptoms are intense, an LTRA is administered in combination with ICSs as controllers. Airway hyper-responsiveness improves or resolves after athletes stop intensive training for a competition." p 319	
Iraq	2012	A	Norma/ near normal	-	-	-	*Recommendation and strategy*<br>"Goals of Management: Maintain normal or near-normal activity throughout the day, including exercise and other physical activities." p 9	*No*
Australia	2019	A + P	Regular	Modera te			*Recommendation*<br>"Risk of reduced bone density should be managed in in patients taking oral corticosteroids (e.g. falls prevention, regular weightbearing exercise and resistance training, adequate calcium and vitamin D intake, anti-osteoporosis treatment where indicated." p 214<br><br>"Regular, moderately intense physical activity improves cardiopulmonary fitness and quality of life in people with asthma, and is well tolerated, but has no effect on lung function or asthma symptoms." p 247<br><br>"There is not enough evidence to recommend one form of physical activity over another in people with asthma. Current evidence does not support the historical belief that swimming is the preferred form of physical training for people with asthma, but few studies have compared effects of swimming with those of other activities: Swimming appears to improve lung function in children and is well tolerated. Overall, swimming does not appear to improve lung function in adults with asthma; but some studies have reported that swimming in non-chlorinated pools improved lung function in adults. Humid air above the surface of swimming pools might be less likely to trigger asthma than dry air environments. However, repeated chlorine exposure over time is associated with chronic airway injury." p 247<br><br>"There is not enough evidence to determine the benefits of other types of exercises, such as tai chi and chi kung (qi gong), in people with asthma." p 247<br><br>*Strategy*	*No*

	"Short-acting beta2 agonists are used to: relieve asthma symptoms, prevent exercise-induced bronchoconstriction, relieve exercise-induced bronchoconstriction." p 28
	"Although cromones are less effective than inhaled corticosteroid in controlling asthma and improving lung function, they may be considered for (any of): • people with symptoms limited to exercise-induced bronchoconstriction." p 61
	"Montelukast taken 1 hour before exercise can be used to manage exercise-induced bronchoconstriction, but it is less effective than short-acting beta2 agonists." p 62
	"Cromolyn sodium and nedocromil sodium taken before exercise can be used to manage exercise-induced bronchoconstriction, but they are only effective in approximately 50% of patients and are less effective than short-acting beta2 agonists." p 62
	"Advise patients that having asthma does not prevent them doing physical activity, including exercise training." p 246
	"Reassure patients that exercise-induced bronchoconstriction can be managed effectively and should not be a reason to avoid physical activity." p 246
	"Safety considerations for physical activity airway injury due to cold air, dry air, or air pollutants (including chlorine in indoor pools) is associated with development of exercise induced bronchoconstriction in elite athletes. Cold air, dry air or air pollutants may also trigger asthma symptoms particularly in athletes." p 247
	"For an adult or child with asthma who has new-onset or worsening symptoms that suggest exercise-induced bronchoconstriction, ask about: the type of physical activity and environment that provokes symptoms timing of symptom onset (symptoms of exercise-induced bronchoconstriction are typically worst 5–10 minutes after stopping exercise, not during exercise) exposure to allergens or other triggers." p 251

	"If history is consistent with exercise-induced bronchoconstriction but other investigations do not demonstrate variable airflow limitation (e.g. spirometry before and 10–15 minutes after bronchodilator shows no or little response), consider referral to a respiratory physician for investigation or referral to an accredited respiratory function laboratory for indirect challenge testing." p 258<br><br>"Laboratory studies show that exercise-induced bronchoconstriction is likely to respond to inhaled corticosteroids if it is associated with airway inflammation and the presence of eosinophils." p 260<br><br>"For an adult with asthma who does not need maintenance inhaled corticosteroid treatment (e.g. mild exercise-induced bronchoconstriction with no symptoms at other times), recommend salbutamol to be taken 15 minutes before exercise. The usual dose range is salbutamol 1–4 puffs via pMDI (100 microg/actuation)." p 264<br><br>"For an adult who experiences exercise-related symptoms on most days and is not already using a preventer, consider daily treatment with an inhaled corticosteroid starting at a low dose. Advise the person to use salbutamol 15 minutes before exercise until the full effect of inhaled corticosteroid has been achieved (usually 2–4 weeks but can be up to 12 weeks)." p 264. "For patients starting inhaled corticosteroid treatment, review efficacy after 4–12 weeks' treatment. If exercise-induced bronchoconstriction has resolved, advise patient to try omitting pre-exercise salbutamol to test whether it is no longer needed." p 266<br><br>"For patients who are taking regular combination inhaled corticosteroid/long-acting beta2agonist treatment and have significant exercise-induced symptoms despite correct inhaler technique and good adherence, consider replacing with inhaled corticosteroid alone as regular maintenance treatment (with as-needed short-acting beta-agonist). A higher dose of inhaled corticosteroid may be needed to maintain good control. Regular montelukast can be used in addition to inhaled corticosteroid.<br>Stopping a long-acting beta2-agonist may cause flare-ups or loss of asthma control. Do not prescribe long-acting beta2-agonists as monotherapy, either intermittently or regularly.

	Montelukast use has been associated with behavioural and/or neuropsychiatric adverse effects, including suicidality." p 266-7<br><br>If exercise-induced symptoms do not resolve after adjusting medicines, and checking adherence and inhaler technique, consider: • alternative diagnoses • referral to an accredited respiratory function laboratory for indirect challenge testing<br>• referral to a respiratory physician for assessment." p 267<br><br>"Advise warm-up before planned exercise." p 267<br><br>"Beta-2 agonists for exercise-induced bronchoconstriction<br>Inhaled beta2-adrenergic receptor agonists are the most effective medicines for short-term protection against exercise-induced bronchoconstriction and for accelerating recovery of lung function after exercise. However, short-acting beta2 agonists should only be taken intermittently (i.e. less than daily), as necessary for preventing exercise induced bronchoconstriction or relieving exercise-induced bronchoconstriction. Daily use of short-acting beta2 agonists may actually increase the severity of exercise-induced bronchoconstriction." p 268<br><br>Beta-2 agonists for exercise-induced bronchoconstriction: doses: Intermittent short-acting beta2 agonists administered by inhalation 5 to 20 minutes before exercise are effective in protecting against exercise-induced bronchoconstriction for 2–4 hours. Salbutamol and terbutaline are equally effective. Recommended doses are as follows: salbutamol 100–400 micrograms by inhalation, 15 minutes before exercise<br>terbutaline 500–1000 micrograms by inhalation, 15 minutes before exercise." p 268<br><br>"Inhaled corticosteroids for exercise-induced bronchoconstriction: Inhaled corticosteroids taken regularly long term (4 weeks or more) are effective in reducing the frequency and severity of exercise induced bronchoconstriction in 30–60% of people with asthma. The degree of protection experienced by individuals ranges from complete to minimal.<br>Patients may need to take inhaled corticosteroid for 12 weeks to experience maximal therapeutic effect. If exercise-induced symptoms have resolved, the

person may no longer need to take a beta2 agonist before exercise. However, some patients taking regular inhaled corticosteroids may still need to take short-acting beta2 agonists before exercise." p 269

Inhaled corticosteroid/long-acting beta-2 agonist combinations for exercise-induced bronchoconstriction: To avoid the possibility of patients taking a long-acting beta2 agonist without an inhaled corticosteroid, long-acting beta2 agonists should (whenever possible) be prescribed as inhaled corticosteroid/long-acting beta2 agonist combination in a single inhaler, rather than in separate inhalers. If no combination product is available for the desired medications, carefully explain to the patient that it is very important that they continue taking the inhaled corticosteroid. Intermittent long-acting beta2 agonists administered by inhalation before exercise are effective in protecting against exercise-induced bronchoconstriction: • for formoterol, onset of bronchodilation and bronchoprotective action is 1-3 minutes after administration • for salmeterol, onset of bronchodilation and bronchoprotective action is 10 - 30 minutes after administration. The duration of effect of both formoterol and salmeterol is up to 12 hours for patients who have not taken a short-acting beta2 agonist or long-acting beta2 agonist within the previous 72 hours. However, the duration of broncho protection is reduced for subsequent doses due to receptor tolerance." p 269

"Montelukast for exercise-induced bronchoconstriction: Montelukast is less effective against exercise-induced bronchoconstriction than short-acting beta2 agonists, but regular use is not associated with receptor tolerance. Montelukast taken either intermittently before exercise or daily is at least partially effective in protecting against exercise-induced bronchoconstriction in some, but not all patients. Some experience strong protection against exercise-induced bronchoconstriction while others experience only partial protection or no effect2 Very few patients experience complete protection against exercise induced bronchoconstriction. The onset of protection occurs within 2 hours of dosing. The duration of protective effect is 12–24 hours. Recommended doses are as follows: • adults 10 mg daily, or 1–2 hours before exercise." p 270

"Cromones for exercise-induced bronchoconstriction: Cromolyn sodium and nedocromil sodium administered by inhalation as single doses before exercise

							partially protect against exercise induced bronchoconstriction in approximately half of patients. The onset of action is rapid. The duration of action is up to 2 hours. Recommended doses are as follows: • nedocromil sodium 4–8 mg by inhalation, 5–10 minutes before exercise • sodium cromoglycate 10–20 mg by inhalation, 5–10 minutes before exercise. Cromolyn sodium and nedocromil sodium are less effective than short-acting beta2 agonists in protecting against exercise-induced bronchoconstriction. However, they have a good safety profile and tolerance does not occur when either of these medicines is taken regularly.” p 270<br><br>“Adjunctive strategies for managing exercise-induced bronchoconstriction: The following strategies may help people with exercise-induced bronchoconstriction manage their symptoms: • warming up before exercise (may enable the athlete to achieve a refractory period) • being as fit as possible – increasing fitness raises the threshold for exercise-induced bronchoconstriction, so that moderately strenuous exercise will not cause an attack • exercising in a warm humid environment - avoiding environments with high levels of allergens, irritant gases or airborne particles • breathing through nose • after strenuous exercise doing cooling down exercise • breathing through the nose and covering the mouth in cold, dry weather • reducing sodium intake Some small clinical trials have suggested that a low-sodium diet might improve lung function after exercise in people with exercise-induced bronchoconstriction, but the clinical importance of this is unknown • fish oil supplementation Some very small, short-term clinical trials reported that fish oil reduced the severity of exercise-induced bronchoconstriction in elite athletes or improve lung function in people with exercise-induced bronchoconstriction, but overall evidence does not support the use of fish oil in asthma • ascorbic acid supplementation A very small, short-term clinical trial reported that ascorbic acid supplementation improved exercise symptoms and asthma control in people with exercise-induced bronchoconstriction, but the clinical importance of this is unknown.” p 270-1	
Canada	2012	A + P	-	-	-	-	-	*No*
Ireland	2015	A	Normal	-	-	-	*Recommendation and strategy*<br>“Goals of long-term management: • Maintain normal activity levels, including exercise.” p 3	*No*

							Strategy<br>"Some forms of exercise, such as running, are more potent triggers. EIB may occur in any climatic condition, but is more common when the patient is breathing dry, cold air and less common in hot, humid climates. Rapid improvement of post-exertion symptoms after inhaled β2-agonists use, or their prevention by pre-treatment with inhaled β2-agonists before exercise, supports a diagnosis of asthma." p 1-2<br><br>"Practical steps to take during unfavourable environmental conditions include avoiding strenuous physical activity in cold weather, low humidity, or high air pollution." p 5	
Malaysia	2017	A	-	-	-	-	*Strategy*<br>"Medication is the mainstay of treatment in EIB. SABA, administered in a single dose before exercise, is effective and safe in preventing EIB. However, long-term regular administration of inhaled β2-agonists induces tolerance and its safety is uncertain due to lack of sufficient data. Warm-up exercise is recommended." p 35<br>"Pharmacological treatment:<br>• Prevent symptoms – SABAs 15 - 20 min pre-exercise<br>• Treat symptoms – SABAs<br>Non-pharmacological treatment (prevent symptoms only)<br>• Warm-up exercise" p 36	*No*
New Zealand	2016	A	-	-	-	-	*Strategy*<br>"Exercise should be encouraged. If exercise provokes asthma it is a marker of poor control and should lead to a review of treatment." p 92	*No*
Oman	2009	A + P	Normal levels	-	-	-	*Recommendation and strategy*<br>"The Initiation and adjustment of asthma therapies is to achieve the followings: Maintain normal activity levels and exercise." p 44<br><br>*Strategy*	*No*

							"This step [SABA] is also recommended as the initial treatment option for patients with bronchospasm on exercise. Recommended: A short-acting inhaled β2 agonist (e.g. Salbutamol inhaler) as required." p 20<br><br>"Exercise-induced Asthma (EIA): Patient should be advised to avoid exercise in cold weather, in places when pollen or air pollution levels are high and to do proper warm-ups before vigorous exercise. Taking short acting β2 agonist 20-30 minutes before exercise is recommended. Leukotriene modifiers should be considered in the management of EIA." p 32	
Poland	2014	A + P	-	-	-	-	-	*No*
Qatar	2016	A	Regular	-	-	-	*Recommendation and strategy*<br>"Lifestyle advice. Offer the following advice: Encourage all people with asthma to exercise regularly." p 26<br><br>"Principles of treatment of chronic asthma: Normalisation of everyday activities. Regular exercise unlimited by disease." p 23<br><br>*Strategy*<br>"For most patients, exercise induced asthma is an expression of poorly controlled asthma and regular treatment including inhaled corticosteroids should be considered. Immediately prior to exercise, inhaled short-acting beta2-agonists are the drug of choice. Treatment with relievers such as short-acting beta2-agonists (SABAs) or anticholinergics, administered 10-15 minutes before exercise is the most preferable method of preventing exercise-induced bronchoconstriction. If exercise is a specific problem in patients taking inhaled corticosteroids who are otherwise well controlled, consider the following therapies **[L2, RGA2]**: Leukotriene receptor antagonists, Long-acting beta2-agonists (LABAs), Theophylline, may be used under the direction of an asthma-specialised physician." p 23	*No*
Saudi Arabia	2019	A + P	-	-	-	-	*Strategy*<br>"It is recommended to avoid outdoor strenuous physical activities in cold weather, low humidity, or high air pollution." p 17	*No*

							"Exercise-induced bronchoconstriction: EIB can be prevented using SABA a few minutes before exercise. A warm-up period before exercise may also reduce EIB symptoms. If this approach does not control the symptoms, the patient is recommended to have maintenance therapy with ICS. Regular use of LTRA may help in this condition, especially in children." p 39<br><br>"LABA provides longer protection to prevent exercise-induced bronchospasm than short-acting inhaled β2-agonists (SABA)." p 69<br><br>"Short-acting bronchodilators, such as salbutamol, are the medications of choice for relief of symptoms of acute attacks of asthma and for the pre-treatment of exercise-induced bronchoconstriction." p 72	
South Africa	2007	A + P	Normal, regular	-	-	-	*Recommendation and strategy*<br>"Set goals of asthma treatment to achieve control: Maintain normal activity levels, including exercise." p 21<br><br>"Strategies to minimise osteoporosis such as regular exercise, calcium supplementation and hormonal replacement in postmenopausal women should be considered." p 22<br><br>*Strategy*<br>"They [LABAs] are useful for control of nocturnal symptoms and exercise-induced asthma. They are recommended as an addition to low dose inhaled corticosteroids in preference to increasing the dose of inhaled corticosteroids (**Evidence A**)." p 22<br><br>"Exercise: The preferred treatment [of EIA] is the use of short-acting inhaled ß2 agonists 15-20 mins before exercise. Long-acting ß2 agonists, leukotriene modifiers and SR theophyllines may protect against EIA for several hours after dosing." p 28	*No*
Spain	2016	A + P	-	-	-	-	*Strategy*<br>"Asthma management **goals**: To prevent daytime, nighttime and exercise-related symptoms. No restrictions on daily life and physical exercise." p 26	*No*

							“Inhaled SABA administered 10-15 minutes before exercise are the drugs of choice to prevent exercise-induced bronchoconstriction.” **(Evidence Level A)** p 27<br><br>“SABAs are the drugs of choice to prevent exercise-induced bronchoconstriction when administered 10 to 15 min before the exercise.” p 34<br><br>“Occasional use of short-acting β2-agonist (SABA) approximately 10 minutes before exercise is the treatment of choice. However, when used regularly, these agents gradually lose efficacy”. **(Evidence Level A)** p 65<br><br>“Increasingly intense warm-up exercise before starting any sports activity may attenuate the intensity of bronchoconstriction”. **(Evidence Level A)** p 65<br><br>“Warm-up exercises before starting any sports activity are recommended, as they may reduce the intensity of bronchoconstriction.” p 68	
Switzerland	2018	A + P	Non-specificPA	-	-	-	*Recommendation*<br>“A number of nonpharmacological strategies and interventions can also be considered to assist with symptom control and risk reduction. They include: physical activity.” p 372	*No*
Turkey	2011	A + P	-	-	-	-	*Strategy*<br>“Rapid-acting inhaled β2-agonists are for relief of bronchospasm during acute exacerbations of asthma and for the pretreatment of exercise-induced asthma”. p 296<br><br>“Leukotriene Receptor Antagonists (LTRA), are mild bronchodilator and anti-inflammatory drugs, used as an alternative treatment in mild persistent asthma, in some patients with aspirin-sensitive asthma and exercise induced asthma. They can be used as addon therapy to reduce the dose of ICS required by patients with moderate to severe asthma.” p 296<br><br>For patients with asthma avoiding physical activity in cold weather and high air pollution are practical recommendations for better control of the disease.” p 298-9	*No*

UK	2019	A + P	-	-	-	yoga/ physical training/ weight loss program	*Recommendation*<br>"Weight-loss interventions (including dietary and exercise-based programmes) should be considered for overweight and obese adults and children with asthma to improve asthma control." **(Evidence level B)** p 58<br><br>"Although current evidence does not support yoga as a routine intervention for people with asthma, it could be considered as an additional therapy or as an alternative to other forms of breathing exercises." **(Evidence rating 1++)** p 60<br><br>*Recommendation and strategy*<br>"As physical training improves indices of cardiopulmonary efficiency, it should be seen as part of a general approach to improving lifestyle and rehabilitation in people with asthma, with appropriate precautions advised about exercise-induced asthma." p 61<br><br>*Strategy*<br>"The aim of asthma management is control of the disease. Complete control of asthma is defined as: no limitations on activity including exercise." p 62<br><br>"Exercise-induced asthma: The following medicines have been shown to give protection against exercise induced asthma:<br>• inhaled corticosteroids; • short-acting β2 agonists; • long-acting β2 agonists; • theophyllines; • leukotriene receptor antagonists; • sodium cromoglicate or nedocromil sodium. Long-acting β2 agonists and LTRA provide more prolonged protection than SABA, but a degree of tolerance develops with LABA particularly with respect to duration of action. No tolerance has been demonstrated with LTRA." p 82 **(Evidence levels varied for each drug)**<br><br>"For most patients, exercise-induced asthma is an expression of poorly controlled asthma and regular treatment including inhaled corticosteroids should be reviewed." p 83<br><br>"If exercise is a specific problem in patients taking inhaled corticosteroids who are otherwise well controlled, consider adding one of the following therapies: • leukotriene receptor antagonists; • long-acting β2 agonists; • sodium	*No*

							cromoglicate or nedocromil sodium; • theophyllines." p 83 **(Evidence levels varied for each drug)**<br><br>"Immediately prior to exercise, inhaled short-acting β2 agonists are the drug of choice." p 83 **(Evidence levels varied for each drug)**	
USA	2007	A + P	Normal	-	-	-	*Recommendation and strategy*<br>"The goals of therapy are to achieve asthma control by (**Evidence A**): Maintain normal activity levels (including exercise and other physical activity and attendance at work or school." p 52 & 227<br><br>*Strategy*<br>"How to control things that make your Asthma worse: Exercise or Sports: • You should be able to be active without symptoms. See your doctor if you have asthma symptoms when you are active—such as when you exercise, do sports, play, or work hard. • Ask your doctor about taking medicine before you exercise to prevent symptoms. • Warm up for a period before you exercise. • Check the air quality index and try not to work or play hard outside when the air pollution or pollen levels (if you are allergic to the pollen) are high." p 130<br><br>"The Expert Panel recommends that clinicians advise patients to avoid, to the extent possible, exertion or exercise outside when levels of air pollution are high (**Evidence C**)." p 176<br><br>"Cromolyn sodium and nedocromil: They can also be used as preventive treatment prior to exercise or unavoidable exposure to known allergens." p 213 & 224<br><br>Cromolyn Sodium and Nedocromil: Indications: • Preventive treatment prior to exposure to exercise or known allergen. Mechanisms: • Inhibits acute response to exercise." p 224<br><br>"LABA may be used before exercise to prevent EIB (**Evidence A**), but duration of action does not exceed 5 hours with chronic regular use. Frequent and chronic use of LABA for EIB is discouraged, because this use may disguise poorly controlled persistent asthma (**Evidence D**)." p 214 & 230	*ICS use may be associated with a dose-dependent reduction in bone mineral content, although low or medium doses appear to have no major adverse effect. Elderly patients may be more at risk due to preexisting osteoporosis, changes in estrogen levels that affect calcium utilization, and a sedentary lifestyle. P 341*

						**“**Zileuton is capable of attenuating bronchoconstriction from exercise.” p 229<br><br>“Short-Acting Beta2-Agonists (SABA). Indications: • Preventive treatment for EIB prior to exercise.” p 247<br><br>“Pretreatment before exercise: • Inhaled beta2-agonists will prevent EIB in more than 80 percent of patients (**Evidence A**)• SABA used shortly before exercise (or as close to exercise as possible) may be helpful for 2–3 hours • LABAs can be protective up to 12 hours. When LABAs are administered on a daily basis, however, there is some shortening of the duration of protection, even in patients using ICSs. Frequent and chronic use of LABAs for EIB should be discouraged. Such use may disguise poorly controlled persistent asthma, which should be managed with daily anti-inflammatory therapy • LTRAs can attenuate EIB in up to 50 percent of patients (**Evidence B**). The onset of action is generally hours after administration. Few comparisons with other protective agents are currently available • Cromolyn or nedocromil taken shortly before exercise is an alternative treatment to prevent EIB, but it is not as effective as SABAs (**Evidence B**) • The addition of cromolyn to a SABA is helpful in some individuals who have EIB. • These studies indicate that anticholinergics may also attenuate EIB, but they are less likely to be protective than either mast cell stabilizers or SABAs • A warmup period before exercise may reduce the degree of EIB (**Evidence C**)• A mask or scarf over the mouth may attenuate cold-induced EIB (**Evidence C**).” p 364	
Caribbean	2009	Keep ‘active’	-	-	-	*Recommendation and strategy*<br>“New approaches to asthma therapy help patients prevent most attacks; remain free of troublesome night and day symptoms and keep physically active. Achieving control of asthma requires: 1. Educating patients and caregivers to manage the condition; 2. Identifying and avoiding triggers that make asthma worse; 3. Selecting appropriate medications; 4. Stopping asthma attacks; 5. Monitoring and modifying asthma care for effective long-term control. Asthma is considered to be controlled if the following criteria are met: No limitation on activities, including exercise.” p 8<br><br>*Strategy*	*No*

	"With your help, and the help of others on the health care team, patients can be actively involved in managing their asthma to prevent problems and can live productive, physically active lives." p 9<br><br>"Do not avoid physical activity. Symptoms can be prevented by taking short- or long- acting inhaled beta-2-agonist or sodium cromoglycate before strenuous exercise." p 13<br><br>"Is the asthma management plan meeting expected goals?<br>Ask the patient: • Are you participating in your usual physical activities? Action to consider: Adjust medications and management plan as needed (step up or step down) after<br>compliance was assessed." p 15

A + P: Adult and Pediatric; SB: sedentary behaviour

Table S2. Recommendations and Strategies (verbatim) pertaining to Sleep from Clinical Practice Guidelines for Asthma.

CPG Country, Year	Types of Strategies Medication	Hygiene	Recommendations Verbatim
Australia, 2019	Increase dose or additional medication		"1. Prescribe an increase in preventer and/or a course of oral corticosteroids for patients with (any of): acute asthma symptoms that recur within 3 hours of taking a rapid-onset beta2 agonist reliever; increasing difficulty breathing over one or more days; night-time asthma symptoms that interfere with sleep over more than one night in a row; peak flow below a pre-defined level (for those monitoring peak flow each day; level determined based on individual's personal best; and history of peak flow levels before and during flare-ups).<br>2. For very uncontrolled asthma at presentation (e.g. frequent night waking, low lung function), consider (either of): high-dose ICS (then down-titrate when symptoms improve) OR a short course of oral corticosteroids in addition to ICS<br>3. Advise patients that if they experience a flare-up (e.g. worsening symptoms over hours or days, or needing reliever again within a few hours), they should increase their reliever use to control symptoms. Include these instructions in the patient's written asthma action plan. More symptoms than usual, increasing difficulty breathing, waking often at night with asthma symptoms."
Caribbean, 2009		Allergens	"1. Remove carpets, especially in sleeping rooms.<br>2. Remove animals from the home, or at least from the sleeping area."
Malaysia, 2017	Increase dose or additional medication		"As-needed reliever therapy: Patients with initial presentation of the following conditions are considered to be managed in Step 1:asthma symptoms OR need for SABA (less than twice a month) AND no waking up at night due to asthma in last month AND no risk factors for exacerbations including no exacerbation in the last year 2) Low dose ICS: The patients with initial presentation of the following conditions are considered to be managed in Step 2: asthma symptoms or need for SABA between twice a month and twice a week OR wakes up at night due to asthma once or more a month with no risk factors in Table 4 • For patients who remain symptomatic in Step 1, treatment should be escalated to Step 2. 3) Low dose ICS/LABA: The patients with initial presentation of the following conditions are considered to be managed in Step 3: troublesome asthma symptoms more than twice a week OR wakes up at night due to asthma once a week or more AND with any risk factors in Table 4 • For patients who remain symptomatic in Step 2, treatment should be escalated to Step 3. 4) Medium/high dose ICS/LABA: Patients with initial presentation of the following conditions are considered to be managed in Step 4: troublesome asthma symptoms more than twice a week AND wakes up at night due to asthma once a week or more AND with any risk factors in Table 4 OR acute exacerbation of asthma requiring hospital admission* • For patients who remain symptomatic in Step 3, treatment should be escalated to Step 4."

Qatar, 2016	Increase dose or additional medication	[redacted]	"Prescribe a regular inhaled corticosteroids at a low dose, in addition to SABA in patients with any of the following: Asthma attack in the last 2 years; Using inhaled beta2 agonists 3 times per week or more.; Symptoms are present 3 times per week or more.; Waking one night per week or more due to asthma symptoms. "
UK, 2019	Increase dose or additional medication	[redacted]	"Inhaled corticosteroids are the recommended preventer drug for adults and children for achieving overall treatment goals. (**Evidence A**) Inhaled corticosteroids should be considered for adults, children aged 5–12 and children under the age of five with any of the following features: using inhaled β2 agonists three times a week or more; symptomatic three times a week or more; or waking one night a week." (**Evidence rating 1+**)
USA, 2007	Increase dose or additional medication	Allergens	"1. Many people who have asthma are allergic to dust mites. Dust mites are like tiny "bugs" you cannot see that live in cloth or carpet. Things that will help the most:<br>- Encase your mattress in a special dust miteproof cover.<br>- Encase your pillow in a special dust mite-proof cover or wash the pillow each week in hot water. Water must be hotter than 130 °F to kill the mites. Cooler water used with detergent and bleach can also be effective.<br>- Wash the sheets and blankets on your bed each week in hot water.<br>Other things that can help:<br>- Reduce indoor humidity to or below 60 percent; ideally 30–50 percent. Dehumidifiers or central air conditioners can do this.<br>- Try not to sleep or lie on cloth-covered cushions or furniture.<br>- Remove carpets from your bedroom and those laid on concrete, if you can.<br>- Keep stuffed toys out of the bed, or wash the toys weekly in hot water or in cooler water with detergent and bleach. Placing toys weekly in a dryer or freezer may help. Prolonged exposure to dry heat or freezing can kill mites but does not remove allergen.<br>2. Avoid sleeping or lying on upholstered furniture.<br>3. To gain more rapid control of asthma, a course of oral systemic corticosteroids may be necessary for the patient who has an exacerbation at the time long-term control therapy is started or in patients who have moderate or severe asthma with frequent interference with sleep or normal activity<br>4. To gain more rapid control of asthma, consider a course of oral systemic corticosteroids for the patient who has an exacerbation at the time long-term controltherapy is started or in patients who have moderate or severe asthma with frequent interference with sleep or normal activity."
South Africa, 2007	Increase dose or additional medication	[redacted]	"1) Treatment with short courses of oral prednisone should be considered in known or newly diagnosed asthmatics in the following circumstances in order to prevent severe deterioration and to gain rapid control of asthma:<br>• Symptoms and/or lung function (PEF) progressively deteriorating acutely or over several days and associated with increased use of inhaled rescue medication<br>• Lack of sustained relief from rescue medication

		[redacted]	• Repeated drops in PEF over 1 or more days to below 60% of previous best value<br>• Frequent night time symptoms<br>• Requirement for emergency treatment”
India, 2005	Increase dose or additional medication	[redacted]	“If the patient has day-time symptoms < 1/week and night-time symptoms ≤ 2/month, then the patient can be managed with reliever medications alone. Use of reliever medications more than 1/week indicates the need for long-term controller medications.”
Iraq, 2012	Increase dose or additional medication	[redacted]	“One daily CONTROLLER medication: Medium-dose inhaled corticosteriod. (50 µgm. 2-4 puffs twice daily) OR Two daily medications: Low-to-medium dose inhaled corticosteroid AND Long-acting bronchodilator especially for nighttime symptoms - sustained-release theophylline or long-acting ß2 agonist (Salmeterol inhaler).”
